# Improvement of endothelial function after switching previously treated HIV-infected patients to an NRTI-sparing bitherapy with maraviroc

**DOI:** 10.7448/IAS.17.4.19726

**Published:** 2014-11-02

**Authors:** Enrique Bernal, Jose Miguel Gomez Verdú, Francisco Vera, Onofre Martinez, Joaquin Bravo, Carlos Galera, Angeles Muñoz, Eva Garcia, Jose Serrano, Ana Perez, Carmen Vera, Irene Marín, Alfredo Cano

**Affiliations:** 1 Infectious Disease Unit, Reina Sofia Hospital, Murcia, Spain; 2 Infectious Disease Unit, Santa Lucia, Cartagena, Spain; 3 Infectious Disease Unit, Morales Meseguer, Murcia, Spain; 4 Infectious Disease Unit, Virgen de la Arrixaca, Murcia, Spain; 5 Cardiology, Reina Sofia Hospital, Murcia, Spain

## Abstract

**Introduction:**

Nucleoside reverse transcriptase inhibitor (NRTI) is associated with endothelial dysfunction and proinflammatory effects. Maraviroc (MVC) is an antagonist of CCR5 receptor. CCR5 is the receptor of RANTES (Regulated on Activation, Normal T Cell Expressed and Secreted), a mediator of chronic inflammation and endothelial function. Our aim was to evaluate the maintenance of viral suppression and improvement of endothelial function in virologically suppressed HIV-infected patients switched to an NRTI-sparing combined antiretroviral therapy (cART) with MVC.

**Materials and Methods:**

This observational, non-interventional, multicenter study was performed at the Infectious Diseases Service of Santa Lucia, Morales Meseguer, Virgen de la Arrixaca and Reina Sofía University Hospital (Murcia, Spain). The selection criteria were to be asymptomatic on a regimen with undetectable viral load (<50 HIV-RNA copies/mL) for at least six months, no previous treatment with R5 antagonists, no evidence of previous protease inhibitor (PI) failure and available R5 tropism test. Twenty-one HIV-infected patients were selected after the treatment regimen was changed to Maraviroc 150 mg/once daily plus ritonavir-boosted PI therapy. Endothelial function was prospectively evaluated through flow-mediated dilatation (FMD) of the brachial artery at baseline and at weeks 24.

**Results:**

We included 21 patients on treatment with PI in combination with 2 NRTI. The mean cART exposition was 133±68.9 months. Fourteen (66.6%) were males, aged 49±9 years, 15 (71.4%) smokers, 4 (19.04%) family history of coronary heart disease, 1 (5.76%) type 2 diabetes and 3 (14.28%) hypertensive, mean total cholesterol was 185.5±35 mg/dL, c-LDL 100.2±37 mg/dL, tryglicerides 170.42±92.03 mg/dL, cHDL 52.6±15.5 mg/dL, CD4 779,5±383.28 cells/mL, nadir CD4 187,96±96 cells/mL. After 24 weeks of follow-up of a switch to an NRTI-sparing regimen, 95.2% of HIV-patients on viral suppressive cART maintained viral suppression and CD4+ T cell count. This cART switch improve endothelial function in patients with lower baseline FMD levels after 24 weeks (baseline FMD −1.19±4.84 % to 24 weeks FMD 11.32±7.27%; p=0.002).

**Conclusions:**

The results of our study show that a switch to an NRTI-sparing bi-therapy with MVC improves endothelial function and maintained the immune-virologic efficacy. This regimen emphasizes the needs for further clinical studies to associate these achievements with the incidence of non-AIDS-defining illnesses.

